# Motor imagery training of goal-directed reaching in relation to imagery of reaching and grasping in healthy people

**DOI:** 10.1038/s41598-022-21890-1

**Published:** 2022-11-03

**Authors:** Joanna Mencel, Jarosław Marusiak, Anna Jaskólska, Łukasz Kamiński, Marek Kurzyński, Andrzej Wołczowski, Artur Jaskólski, Katarzyna Kisiel-Sajewicz

**Affiliations:** 1 Department of Kinesiology, Faculty of Physiotherapy, Wroclaw University of Health and Sport Sciences, Al. I. J. Paderewskiego 35, budynek P4, 51-612 Wrocław, Poland; 2 Department of Field Theory, Electronic Circuits and Optoelectronics, Faculty of Electronics, Photonics and Microsystems, Wroclaw University of Science and Technology, Wroclaw, Poland

**Keywords:** Brain-machine interface, Motor neuron, Premotor cortex

## Abstract

The study aimed to determine whether four weeks of motor imagery training (MIT) of goal-directed reaching (reaching to grasp task) would affect the cortical activity during motor imagery of reaching (MIR) and grasping (MIG) in the same way. We examined cortical activity regarding event-related potentials (ERPs) in healthy young participants. Our study also evaluated the subjective vividness of the imagery. Furthermore, we aimed to determine the relationship between the subjective assessment of motor imagery (MI) ability to reach and grasp and the cortical activity during those tasks before and after training to understand the underlying neuroplasticity mechanisms. Twenty-seven volunteers participated in MIT of goal-directed reaching and two measurement sessions before and after MIT. During the sessions 128-channel electroencephalography (EEG) was recorded during MIR and MIG. Also, participants assessed the vividness of the MI tasks using a visual analog scale (VAS). The vividness of imagination improved significantly (*P* < .05) after MIT. A repeated measures ANOVA showed that the task (MIR/MIG) and the location of electrodes had a significant effect on the ERP's amplitude (*P* < .05). The interaction between the task, location, and session (before/after MIT) also had a significant effect on the ERP's amplitude (*P* < .05). Finally, the location of electrodes and the interaction between location and session had a significant effect on the ERP's latency (*P* < .05). We found that MIT influenced the EEG signal associated with reaching differently than grasping. The effect was more pronounced for MIR than for MIG. Correlation analysis showed that changes in the assessed parameters due to MIT reduced the relationship between the subjective evaluation of imagining and the EEG signal. This finding means that the subjective evaluation of imagining cannot be a simple, functional insight into the bioelectrical activity of the cerebral cortex expressed by the ERPs in mental training. The changes we noted in ERPs after MIT may benefit the use of non-invasive EEG in the brain-computer interface (BCI) context.

Trial registration: NCT04048083.

## Introduction

Motor imagery (MI) is the conscious, mental simulation of action without body movement^1^. Imaging studies have indicated that MI and execution of movement share overlapping brain areas, namely: the primary motor cortex, supplementary motor area, superior and inferior parietal lobules, dorsal and ventral premotor cortices, prefrontal cortex, inferior frontal gyrus, superior temporal gyrus, sensory cortex, anterior cingulate gyrus, basal ganglia, and cerebellum^2–9^. In addition, many studies have shown that motor imagery training (MIT) may facilitate motor skill learning^10–12^ and neurorehabilitation^13–16^. Therefore, further research into its mechanisms for optimal use was recommended^17,18^.

MI is also important in the brain-computer interface (BCI) field^11,19,20^. BCIs acquire signals from the brain and analyze and translate them into commands relayed to output devices or neuroprostheses that carry out the desired movements^21,22^. Electroencephalography (EEG) is a non-invasive recording of brain signals used for BCI. The power modulation of the sensorimotor rhythm (SMR) recorded via EEG during MI has been used for BCI. The SMR ranges from 8 to 12 Hz, often with beta (about 20 Hz) and gamma (about 40 Hz) components, and is recorded above the sensorimotor cortex. The SMR desynchronizes with movement execution or imagery, referred to as event-related desynchronization (ERD). It also synchronizes after movement execution or imagery, that is, event-related synchronization (ERS)^23^.

Recently, attention has been paid to using time-domain analysis of the EEG signal^24,25^. The latter method does not require BCI users to learn unnatural MI commands. Such commands include using the repetitive imagination of foot movements to control hand functions or MI of the left hand to control right-hand functions^26^, as in the case of SMR-based BCI. Analyzing the EEG signal in the time domain with MI tasks allows for characterizing event-related potentials (ERPs), also called motor-related cortical potentials (MRCPs). These are often phase- and time-locked to the physical execution of movement cortical potentials^27,28^ rather than their imagination. Previous studies have shown that ERP is highly specific for motor-related processes^12,29,30^. In the ERP waveform, several components can be identified at identified at relevant scalp topographic regions of interest. They provide information about sensory perception processes with a short latency after a stimulus (exogenous components) and/or higher-level processing such as cortical inhibition, attention, response selection, memory update-related processes, and other cognitive activity (endogenous components)^31^. Recent studies also indicate an important role for the N2/N2-like component in human executive functions^32^. This component of the ERP is usually observed at medial-frontal sites between 200 and 400 ms after stimulus^33^. In addition, the N2 component was shown to reflect ERP changes during physical^34^ and mental training^35^.

In neurorehabilitation and among end users of BCIs, MI is essential for upper limb function. Hand function is a top priority for people with tetraplegia^36^ or with spinal cord injury^37^, and most activities of daily living are based on goal-directed reaching^25^. This movement is also consistent with modern goal-oriented practice in neurorehabilitation. The practice relies more on the reeducation of function than on training individual muscles or movement in a single plane^38^.

In goal-directed reaching, two components are distinguished, namely reaching and grasping. According to the classical approach, they are non-sequentially programmed in parallel by separate parieto-premotor pathways^39^. Those different pathways are responsible for the sensorimotor transformation process based on visual information about the target location in relation to the body, the upper limb position for reaching, and the information about the object, such as the dimensions and shape, for the grasping component^39^. The study by Glover et al.^40^ has also indicated that distinct networks of the human cortex control programming and the online controlling of reaching-to-grasp movement assessed by functional magnetic resonance imaging (fMRI). They have shown that the planning network includes the premotor cortex, basal ganglia, anterior cingulate, medial parietal area, superior parieto-occipital cortex, and middle intraparietal sulcus. The sensorimotor cortex, cerebellum, supramarginal gyrus, and superior parietal lobule are involved in the control network. On the one hand, it is known that, cortical regions involved in MI tasks change with experience^41^. On the other hand, the study by Ofner et al.^24^ has shown that upper limb movements can be decoded from the ERPs, but ERPs related to movement execution are more pronounced for movement execution than MI tasks. Ofner et al.^24^ suggested that MIT could be highly beneficial for ERPs-based classification during MI tasks.

Excluding our preliminary study of one person born without upper limbs and a healthy control^42^, we did not find evidence about MIT of goal-directed reaching and cortical activity associated with motor imagery of reaching (MIR) and motor imagery of grasping (MIG). We also decided to check the relationship between EEG and subjective vividness of MIR and MIG tasks after the study by Zabicki et al.^43^ showed that spatial patterns of neural activity within the human motor cortices assessed by fMRI reflect the individual vividness of imagined actions.

Therefore, this basic research aimed to determine whether four weeks of MIT of goal-directed reaching (reaching-to-grasp task) would affect the cortical activity during MIR and MIG in the same way in terms of ERPs in healthy young participants. In addition, we aimed to assess the task imagery vividness and its correlation with the EEG signal.

We assumed that the bioelectric activity of the cerebral cortex related to MIR and MIG would change after MIT and that MIR and MIG would vary. We also assumed that the effect of MIT would differ for the two tasks and that activity would vary in different areas of the cerebral cortex. Additionally, we hypothesized that there is an association between the bioelectric activity of the cerebral cortex related to MI and the subject's perceived vividness of the image. Finally, we predicted that the strength of the correlation between the subjective vividness of MI tasks and EEG signal would change after four weeks of MIT.

Our study extends the knowledge about the mechanisms of MIT of reaching-to-grasp movement concerning motor commands of its two components according to the classical approach to the neural control of this movement^39^. In goal-oriented practice, it also contributes to the optimal use of MIT for essential upper limb functions in neurorehabilitation. The results may be helpful in the context of BCI, although this study does not directly address methodological issues in this field.

## Material and methods

### Subjects

Twenty-seven (12 women and 15 men) healthy volunteers (age: 25 ± 3 years; height: 1.72 ± 0.09 m; and body mass: 71 ± 13.8 kg) participated in this experiment. They were right-handed according to the Edinburgh handedness inventory^44^. They also had a moderate ability to kinesthetically imagine motor tasks assessed by the Motor Imagery Questionnaire-Revised Second version (32 ± 8). Note that the minimum value for this modality is seven, and the maximum is 49 in this questionnaire^45^). Furthermore, the volunteers had no motor or neurological impairments. Our study included subjects with an average ability to imagine motor tasks and subjects who did not have experience in such training. This decision reflects the finding that the effectiveness of MIT is related to a subject's motor imagination capability. That is, its effectiveness is higher for more vivid imagery^46^) and with the subjects' experience with MIT^41^. This study sample mimics future patients.

**Figure 1:**
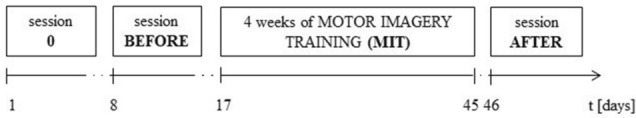
Scheme of the experimental protocol, consisting of familiarization session 0 and two similar measurement sessions before and after motor imagery training.

All subjects provided written informed consent before taking part in this experiment. The ethics committee of the Wroclaw University of Health and Sport Sciences in Poland approved the experimental protocol. The study was carried out according to the World Medical Association Declaration of Helsinki—Ethical Principles for Medical Research Involving Human Subjects (2013). All subjects participated in a short familiarization session, two similar measurement sessions (before and after training), and four weeks of MIT (Fig. 1). This study is a part of a registered experiment (ID: NCT04048083; https://clinicaltrials.gov/ct2/show/NCT04048083?term=NCT04048083&draw=2&rank=1).

### MIT of reaching to grasp a book

The participants participated in 12 training sessions held thrice per week for four weeks of kinesthetic motor imagery training of goal-directed reaching (reaching to grasp a book) when comfortably seated in front of a desk. There were two one-day breaks and one two-day break between training sessions. We implemented the kinesthetic modality of MIT. Most authors agree that this approach is more effective compared to the visual approach^15^, more markedly reorganizes sensorimotor networks^47^, and produces higher levels of physiological responses^30^. The training protocol was designed according to the above recommendation^48^ and the Physical, Environment, Task, Timing, Learning, Emotion, and Perspective (PETTLEP) model^49^.

**Figure 2:**
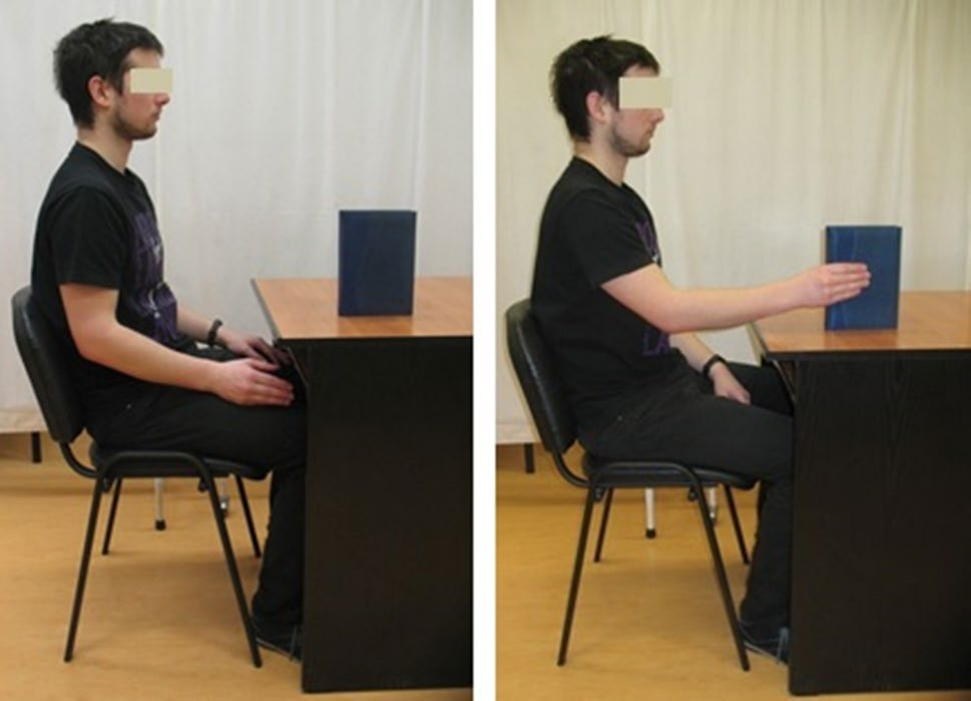
Position of the participant during motor imagery training of reaching to grasp a book task. The participant performed this movement physically three times with the right and left upper limbs before beginning mental rehearsals, paying attention to the kinesthetic sensations that accompany this movement. Written informed consent for publication of the images was obtained from the participant.

At the beginning of each training session, the subjects performed three physical executions of reaching to grasp a book with the right upper limb and three physical executions of this movement by the left upper limb before the non-dominant upper limb part of the training. The same book was used in each session for all participants. This part of the training session introduced the participants to the location and features of the book (such as size, weight, surface roughness, and fragility) used by the central nervous system to program this goal-directed movement^37^. The location was always the same distance from the participant and was similar for all participants. It required flexion at the shoulder joint to an angle of about 30° to grasp the book (Fig. 2). The book measured 14.8 by 21.0 by 2.5 cm and weighed 0.497 kg, with a dark blue leather cover (Fig. 2). A whole-hand-lumbrical grasp of a book placed vertically on a desk was used. It involved keeping the fingers straight (Fig. 2).

During the movement execution trials, the instructor asked that attention be paid to the kinesthetic sensations accompanying the movement. The instructor pointed out, in turn, the stretching of muscles, changes in joint angles, the varying sensation of weight on the limb as the movement progressed toward the book, and the feeling of grasping a book placed vertically on a table in front of the participant with sufficient force to lift it subtly. Lifting of the book was not imagined, this information was only to help determine the amount of grasp force generated. It should be noted that the order of the kinesthetic sensations refers to reaching and grasping corresponding to the natural course of this movement. Then, the book was taken, and participants were instructed to relax and not perform any movements or muscle contractions during mental training.

After a short break, MIT started. Following the instructor's command of 'attention,' the subjects closed their eyes and waited. After the command of 'start' from the instructor, the trial began. During each trial, the subjects were asked to recall their kinesthetic sensations of functionally reaching to grasp the book indicated above. The opening of the subject's eyes marked the end of the trial, and the instructor began measuring the 20-s break. After 15 s, the instructor issued the command of 'attention.' Then, the subjects closed their eyes, and another trial began. Sensors did not objectively measure eye opening and closing. The instructor observed them. Each training session consisted of 10 trials in three sets, performed separately with the right and left upper limbs. That is ten trials of three sets for the two upper limbs. The trials with the right upper limb were done first, followed by the left. There were 20 s of rest between trials, a three-minute rest between sets, and a 15-min rest between the right and left upper limbs. During one training, the participants imagined reaching to grasp a book 30 times with the right upper limb (10 trials × 3 sets) and 30 times (10 trials × 3 sets) with the left upper limb. One training session took between 50 and 52 min, depending on the individual's MI time.

### Measurement sessions

During the familiarization session (session 0, Fig. 1), the subjects could see where the research would be conducted, weight and height measurements were made (Seca, Germany), and the Edinburgh handedness inventory^40^ was obtained. Then, the subjects completed the Motor Imagery Questionnaire-Revised Second version^45^. The measurement sessions before and after training were conducted the same way and took place in the morning on two consecutive days. On the first day of both sessions, the participants imagined reaching 20 times, that is, MIR. After a short break, they imagined a whole hand, lumbrically grasping the book used during MIT 20 times with the dominant and non-dominant upper limbs, that is, MIG. They undertook MIR for the right upper limb, MIG for the right hand, MIR for the left upper limb, and MIG for the left hand, in the same orderduring both measurement sessions. During the tasks, EEG and electromyography (EMG) signals were recorded. This study did not include tasks for the non-dominant upper limb. Instead, we analyzed the dominant upper limb after Gentili and Papaxanthis^50^ demonstrated the superiority of mental training of the reaching movement for the dominant compared to the non-dominant upper limb. In their study, the mental training resulted in greater and more robust improvements in the movement performance for the dominant compared to the non-dominant limb.

On the second day, in both measurement sessions (before and after), the subjects executed reaching and grasping. However, this data is not presented as the output signals' analysis goes beyond this article's aims.

During the MIR and MIG tasks, the subjects were seated comfortably with their eyes open. Their first task was to imagine kinesthetically reaching with the right upper limb for a book (MIR). Then, they imagined kinesthetically grasping the book (MIG). MIR and MIG were repeated 20 times each to avoid fatigue and achieve a sufficient number of trials for the primary analysis (averaging the data). During the training, the subjects imagined the functional movement of reaching to grasp the book in a kinesthetic context. This process involved reaching and grasping the book with force, allowing it to be gently lifted, which was not imaginary. During the measurement sessions, they separately imagined the movement of reaching or transporting the upper limb at the same distance as during training but without grasping a book (MIR). They then imagined grasping it with force, allowing it to be subtly lifted (MIG). The participants were given clear and similar instructions. In the reaching task, they were asked to focus on the kinesthesia involved in reaching or transporting the limb. The kinesthesia involved stretching muscles, changes in joint angles, the varying sensation of weight on the limb as the movement progressed, and maintaining a whole hand lumbrical grasp position. In the grasping task, they were asked to focus on the kinesthesia of grasping the book. The kinesthesia involved the sensation of grasping a book placed vertically on a table in front of the participant and the amount of mentally-generated grasping force. This process corresponds to the hypothetical situation of discrete lifting, which was not imagined. This separation of goal-directed reaching movement into two components was emphasized by the information about the separate movements they performed during the measurement sessions. As mentioned above, participants performed reaching and grasping separately on the second day of measurements. However, they received preliminary information about this on the first day.

To verify the vividness of the MI tasks achieved, immediately after 20 repetitions of MIR and 20 repetitions of MIG, the participants marked a horizontal line on a 10 cm visual analog scale (VAS), from 0 (very easy to feel) to 10 (very hard to feel). Therefore, this assessed the vividness of kinesthetic sensation ('feel') and not the tasks' visualization ('see').

### EEG and EMG data recording

EEG data were recorded continuously using Ag/AgCl electrodes from 128 scalp electrodes using the Biosemi Active-two system (Biosemi Inc., Amsterdam, the Netherlands). The electrodes were clipped in a nylon electrode cap in holders placed according to the Biosemi-designed equiradial system, partially overlapped with an extended International 10–20 system^51^. The EEG signals were amplified with a bandpass of 0–128 Hz and sampled at 2048 Hz. The EEG data recording did not begin until the impedance for all electrodes settled below five kΩ. Subjects were instructed not to move their body and head or blink their eyes. These were allowed between trials and during breaks. At the same time, bipolar surface EMG (Biosemi, Inc., The Netherlands) signals from the anterior part of the deltoid, abductor brevis, and the first dorsal interosseous muscles of the right and left upper limbs were recorded during MIR and MIG tasks. This aimed to control the activation of these muscles that should be withheld in MI. EMG signals were analyzed in Spike2 software (Spike2, CED, Cambridge, UK). Windows of eight seconds from the trial durations (not breaks) of MIR and MIG were used for the analysis. In addition, an electrical recording of acoustic signals (single to start the trial and double to stop it) on a separate channel was used to determine the windows for the analysis. A root-mean-square (RMS) value exceeding two standard deviations (SD) measured from baseline indicated muscle activation. It was not necessary to remove any trials from further analysis because muscle activation was not noted.

### EEG data processing and analysis

The EEG signal data were analyzed using the Brain Electrical Source Analysis 7.0 software (BESA, MEGIS Software GmbH, Gräfelfing, Germany). During offline processing, the EEG signal was down-sampled to 512 Hz (Decimator, Biosemi Inc.), inspected visually, bandpass filtered between 0.53 and 50 Hz, and a notch filter at 50 Hz was used. Parts of the EEG signals containing artifacts such as blinking or biting were cut off. Then, the EEG signals associated with each subject's MIR and MIG tasks were trigger-averaged using a single tone. For this purpose, individual voltage maps were first visually evaluated, and the longest imagining time was estimated. Based on this step, we used a 2000 ms window for averaging, where 0 was the time of the single tone and indicated that the subject should start the MIR or MIG task. The 100 ms interval was used for baseline correction (from −100 to 0 ms). Notice that the MRCP can be time-locked to the physical execution of a tested movement when its actual onset (for example, a specific increase in EMG activity of an agonist muscle) is used as a temporal trigger for the analysis. However, using such an individual temporal trigger in imagining movement tasks is impossible as there is no objective output signal. Another difficulty is that the voltage maps for MI are more dispersed than those associated with physical motor performance. Therefore, we chose an external auditory cue, similar to others^30^, for averaging purposes. For the sessions held before and after, the mean values ± SD free from artifacts trials taken for averaging were: 17.60 ± 1.99 and 17.73 ± 1.62 for the reaching task and 18.47 ± 1.75 and 17.21 ± 1.70 for the grasping task. In the main analysis, we targeted an ERP negative component (N2-like), as previous studies indicated that it is sensitive to ERP changes due to physical and mental training^34,35^.

ERP amplitude (the peak value of N2-like component [µV]) and its time point of occurrence relative to the trigger used (ERP latency [ms]) were calculated for 12 electrodes of interest located above the motor-related regions: F3, Fz, F4, FC3, FCz, FC4, C3, Cz, C4, CP3, CPz, and CP4. This step was done semiautomatically, excluding the 0–100 ms time interval, which corresponds to exogenous potentials^31^, especially in the context of the auditory trigger. The electrodes F3 and FC3 were located above contralateral to the tasks' premotor cortex. In contrast, the F4 and FC4 were above ipsilateral to the tasks' premotor cortex. Other electrodes were located centrally or centrally-parietal. The electrodes C3 and CP3 were located above the sensorimotor cortex contralateral to the tasks, the C4 and CP4 electrodes were located above the sensorimotor cortex ipsilateral to the tasks, and the Cz and FCz electrodes corresponded to the supplementary motor area (SMA). We report the results below on the MIR and MIG with the dominant upper limb. Therefore, information about the side will be omitted for clarity of the text. The right hemisphere means ipsilateral to the tasks, and the left hemisphere means contralateral to the tasks.

### Statistical analysis

The sample size was calculated a priori in the G*Power tool^47^ to achieve a power of the assumed statistical tests of more than 0.8 with the significance level set at α ≤ 0.05. As a result of this calculation, 27 participants were recruited for the study.

Friedman repeated measures analysis of variance (ANOVA) by ranks, and follow-up comparisons were used to assess potential differences between sessions (before vs. after training) and tasks (MIR vs. MIG) in the vividness of motor imageries. Distribution-free methods are recommended for VAS analysis^53^.

The Shapiro–Wilk test verified the normality of EEG data distribution. Based on the result of this test (41 of 48 for ERP amplitude and 30 of 48 for ERP latency met the normality criterion, *P* > 0.05), we undertook a repeated measures ANOVA of the EEG signal (separately for the ERP amplitude and ERP latency parameters). The following factors were incorporated into this analysis: two sessions (before and after), two tasks (MIR and MIG), and 12 (location of electrodes). As indicated by Mauchly's test, the Greenhouse–Geisser correction was applied when sphericity was violated. The partial eta-squared (${\upeta }_{\mathrm{P}}^{2}$) test was used as a measure of effect size for a repeated measures ANOVA. Post hoc comparisons were made for statistically significant factors indicated by ANOVA.

In addition, a non-parametric Spearman's rank correlation analysis was performed (based on the ordinal character of the VAS scale data^53^) to assess the relationship between the EEG signal (separately for individual electrodes and the signal's parameters) and the subjective vividness of MIR and MIG for both measurement sessions (before and after). The strength of the correlation was culled from Hair^54^.

The test results were considered statistically significant when *P* ≤ 0.05. Data were analyzed using SPSS 22.0 software (IBM, Armonk, NY, USA).

### Ethics approval and consent to participate

All participants provided written informed consent before taking part in this experiment. The experimental protocol was approved by the ethics committee of the Wroclaw University of Health and Sport Sciences in Poland and the study was carried out according to the standards set out in the World Medical Association Declaration of Helsinki (2013).

## Results

### Vividness of MI (subjective vividness rating)

**Figure 3:**
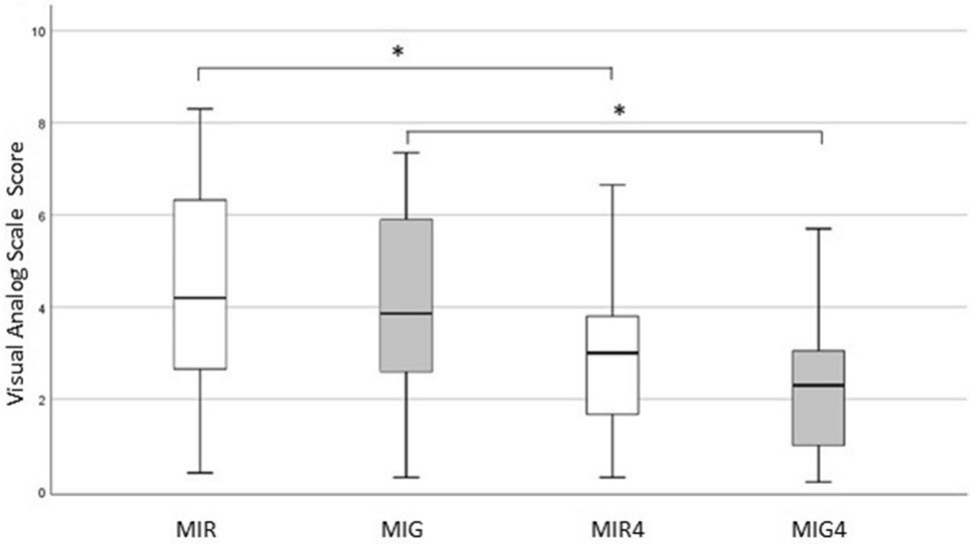
Boxplot representing median values, 25–75% range (box), and min–max range (bars) of vividness expressed by visual analog scale (VAS) score (y-axis) of motor imagery of reaching (MIR) and motor imagery of grasping (MIG) before and after four weeks of training (MIR4, MIG4 respectively). The lower number, the higher the vividness*—*P* = 0.002 (assessed by Wilcoxon signed-rank test).

The vividness of MIR and MIG was significantly higher (*P* = 0.002 for both) after four weeks of MIT (VAS-MIR4 and VAS-MIG4 respectively; Fig. 3), based on post hoc analysis, after *P* < 0.001 in Friedman repeated measures analysis of variance by ranks (χ^2^(3) = 21.739, *P* < 0.001). However, there was no significant difference between the vividness of the two imaginary tasks before (*P* = 0.55) and after training (*P* = 0.071, Fig. 3) as assessed by the Mann–Whitney U test.

### ERP amplitude

**Figure 4:**
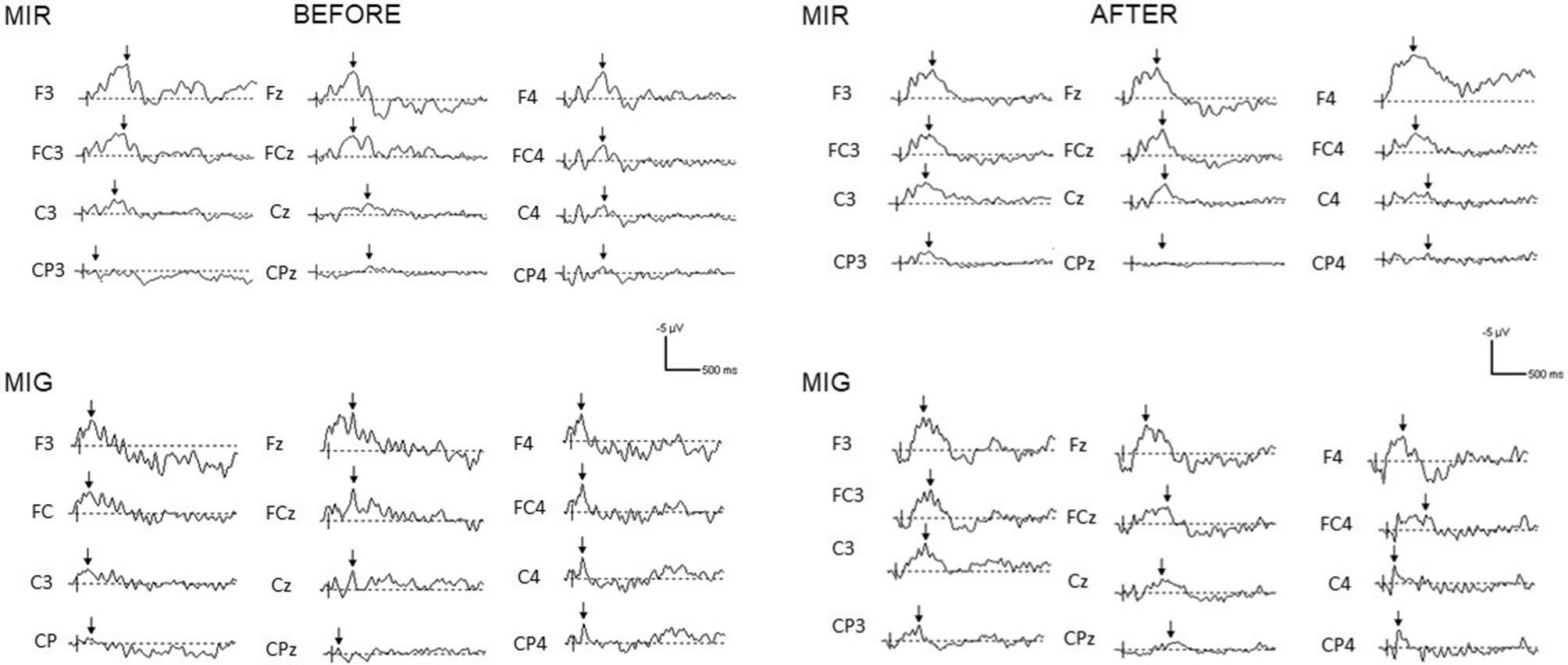
Individual patterns of ERP during motor imagery of reaching (MIR) and motor imagery of grasping (MIG) for chosen electrodes and measurement sessions (before and after). The vertical lines indicate the time of occurrence of the trigger, and the arrows indicate the ERP amplitude.

Individual patterns of ERPs during MIR and MIG for both measurement sessions are shown in Fig. 4. Table 1 presents mean values with SD of ERP amplitudes for the electrodes of interest (Table 1). A repeated measures ANOVA shows that the task and location of electrodes, as well as interaction between session, task, and electrode location, had a significant impact (*P* < 0.05) on the ERP amplitude (Table 2). However, the session and other interactions between factors (Table 2) had no significant effect (*P* > 0.05) on the ERP amplitude.

**Table 1:** Mean value with standard deviation of ERP amplitude [µV] for motor imagery of reaching and motor imagery of grasping before and after 4 weeks of motor imagery training. Notice that the spatial arrangement of the electrodes in the table corresponds to the International 10–10 System.

Before training						After training					
Electrode	Mean ± SD [µV]	Electrode	Mean ± SD [µV]	Electrode	Mean ± SD [µV]	Electrode	Mean ± SD [µV]	Electrode	Mean ± SD [µV]	Electrode	Mean ± SD [µV]
Motor imagery of reaching											
F3	−8.04 ± 1.93	Fz	−8.44 ± 2.64	F4	−7.43 ± 2.39	F3	−8.42 ± 4.25	Fz	−9.90 ± 3.59	F4	−8.03 ± 2.92
FC3	−5.31 ± 1.42	FCz	−7.12 ± 2.28	FC4	−6.01 ± 2.72	FC3	−5.50 ± 1.57	FCz	−8.75 ± 2.57	FC4	−6.18 ± 2.82
C3	−4.25 ± 1.45	Cz	−4.93 ± 1.80	C4	−4.48 ± 2.14	C3	−4.58 ± 1.79	Cz	−5.77 ± 1.55	C4	−5.29 ± 2.62
CP3	−3.44 ± 1.15	CPz	−2.44 ± 1.01	CP4	−4.18 ± 2.07	CP3	−3.59 ± 1.10	CPz	−2.42 ± 0.85	CP4	−4.15 ± 1.54

Electrode	Mean ± SD [µV]	Electrode	Mean ± SD [µV]	Electrode	Mean ± SD [µV]	Electrode	Mean ± SD [µV]	Electrode	Mean ± SD [µV]	Electrode	Mean ± SD [µV]
Motor imagery of grasping											
F3	−6.76 ± 2.02	Fz	−8.19 ± 3.10	F4	−7.55 ± 2.63	F3	−8.11 ± 4.21	Fz	−7.97 ± 3.68	F4	−6.66 ± 3.02
FC3	−5.49 ± 2.12	FCz	−7.05 ± 2.18	FC4	−5.71 ± 1.91	FC3	−5.32 ± 2.08	FCz	−8.00 ± 3.31	FC4	−5.04 ± 2.08
C3	−3.99 ± 1.40	Cz	−4.77 ± 1.29	C4	−4.49 ± 1.70	C3	−4.46 ± 1.73	Cz	−5.20 ± 1.81	C4	−3.72 ± 1.54
CP3	−2.91 ± 1.03	CPz	−2.21 ± 0.80	CP4	−3.93 ± 1.74	CP3	−3.66 ± 2.60	CPz	−2.64 ± 1.30	CP4	−4.28 ± 2.27

**Table 2:** The results of repeated measures ANOVA for ERP amplitude parameter.

Factor	*F* value	*P* value	${\upeta }_{\mathrm{P}}^{2}$
Session	*F*(1, 26) = 2.477	*P* = 0.128	0.087
Task	*F*(1, 26) = 5.780	***P* = 0.024**	0.182
Location of the electrodes	*F*(4.118, 107.066) = 73.126	***P* < .01**	0.738
Session x task	*F*(1, 26) = 0.702	*P* = 0.410	0.026
Session x location	*F*(4.746, 123.392) = 1.651	*P* = 0.155	0.006
Task x location	*F*(4.802, 124.840) = 1.206	*P* = 0.311	0.044
Session x task x location	*F*(5.026, 130.675) = 2.355	***P* = 0.044**	0.083

Significant are in bold.

#### ERP amplitude for two tasks (MIR vs. MIG)

**Figure 5:**
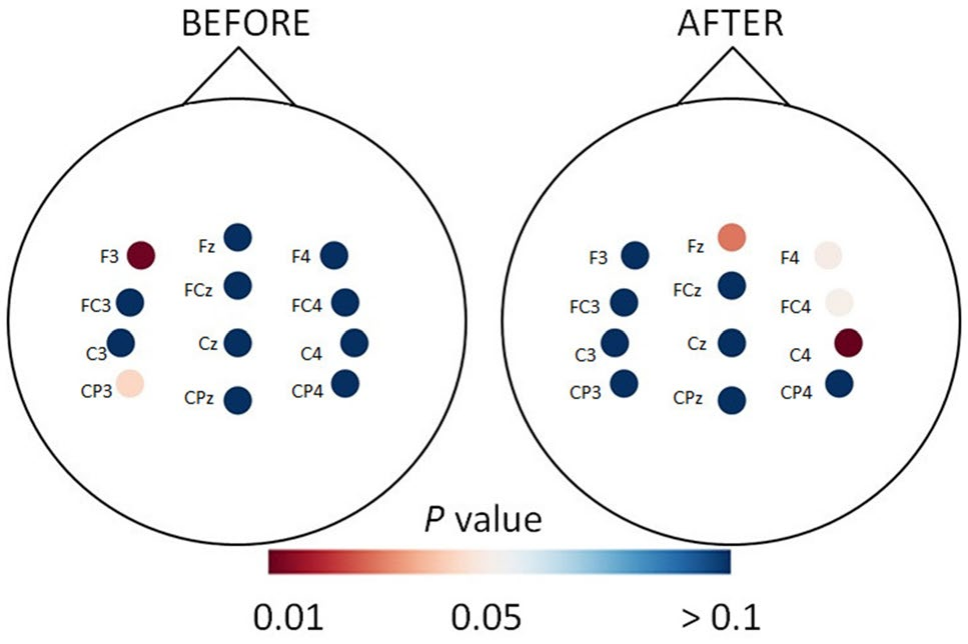
Heatmap depicting the *P* values of pairwise comparison for ERP amplitude between motor imagery of reaching (MIR) and motor imagery of grasping (MIG) before and after four weeks of motor imagery training.

Pairwise comparisons show that ERP amplitudes for MIR were significantly higher (*P* < 0.05) compared to those for MIG for two of 12 electrodes before training (F3: *t*(52) = −2.35, *P* = 0.011, d = 0.01, and CP3: *t*(52) =  −1.76, *P* = 0.04, d = 0.04) and four electrodes after training (Fz: *t*(52) =  −1.94, *P* = 0.02, d = 0.05, F4: *t*(52) =  −1.70, *P* = 0.04, d = 0.04, FC4: *t*(52) =  −1.69, *P* = 0.04, d = 0.04 and C4: *t*(52) =  −2.67, *P* = 0.005, d = 0.07, Fig. 5). There were no significant differences between other pairs of electrodes (*P* > 0.05).

#### ERP amplitude for different electrode locations

**Figure 6:**
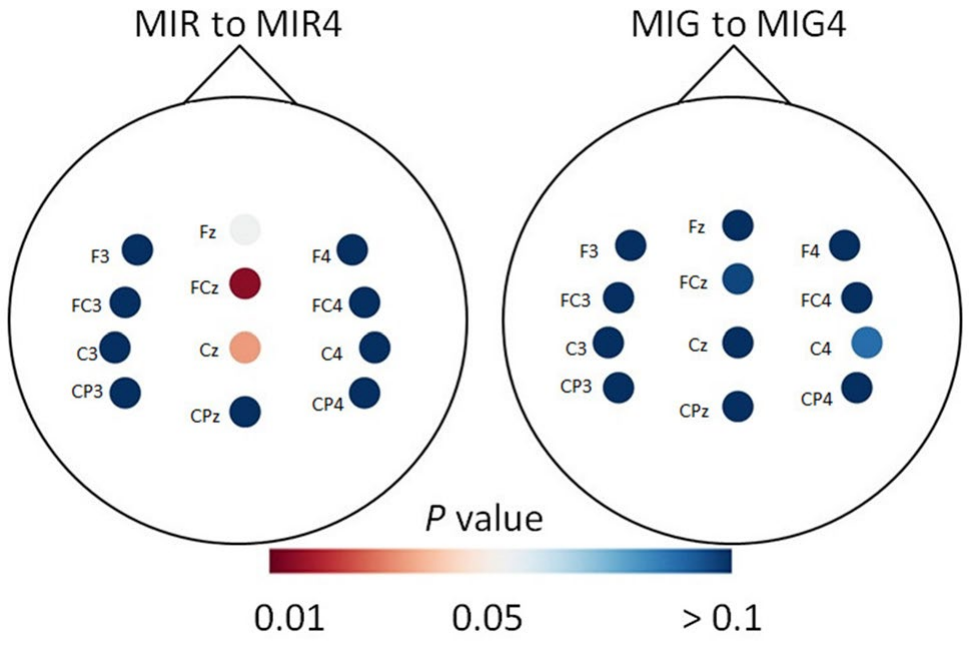
Heatmap depicting the *P* values of pairwise comparison for ERP amplitude between measurement sessions (before and after training) for motor imagery of reaching (MIR to MIR4) and motor imagery of grasping (MIG to MIG4).

We dropped the comparison of all electrodes in favor of functionally meaningful comparisons. This solution reduced the number of comparisons from 66 to 8. Therefore, four pairwise comparisons were performed between electrodes above the left and right hemispheres: F3 vs. F4, FC3 vs. FC4, C3 vs. C4, and CP3 vs. CP4 (left to right; shaded area in Table 3). Also, four pairwise comparisons were performed between electrodes located above the premotor cortex and the sensorimotor cortex of the same hemisphere: F3 vs. C3, FC3 vs. CP3, F4 vs. C4, and FC4 vs. CP4 (top-to-bottom; Fig. 6). The result shows that for the left to right comparisons, there were no significant differences (*P* > 0.05) between electrodes for both tasks and sessions, except for the higher ERP amplitude for CP4 compared to CP3 for MIG during the session before training (*P* = 0.012; Table 3). However, for the top-to-bottom analysis, all the electrodes located above the premotor cortices had significantly (*P* < 0.05) higher ERP amplitudes than the electrodes above the sensorimotor cortices for both tasks and sessions, except FC4 vs. CP4 for MIG and the session after training (Table 3).

**Table 3:** Results of pairwise comparison for ERP amplitude between electrodes located above the right compared to the left hemisphere, and between electrodes located above the premotor cortex compared to the sensorimotor cortex of the same hemisphere for MIR and MIG before and after MIT assessed with independent *t*-*test*. Bold font indicates statistical significance.

Motor imagery of reaching							
Before					After		
Pair comparison	*df*	t	*P*	d	t	*P*	*d*
F3 vs. F4	52	1.01	0.314	0.27	0.39	0.697	0.10
FC3 vs. FC4	52	−1.18	0.242	0.33	−1.10	0.276	0.31
C3 vs. C4	52	−0.44	0.660	0.12	−1.16	0.249	0.32
CP3 vs. CP4	52	−1.62	0.111	0.45	−1.52	0.133	0.42
F3 vs. C3	52	−8.10	** < .001**	2.22	−4.33	** < .001**	1.27
FC3 vs. CP3	52	−5.29	** < .001**	1.45	−5.14	** < .001**	1.42
F4 vs. C4	52	−4.78	** < .001**	1.30	−3.62	** < .001**	0.98
FC4 vs. CP4	52	−2.77	**0.008**	0.76	−3.27	** < .002**	0.92

Motor imagery of grasping							
Before					After		
Pair comparison	*df*	t	*P*	d	t	*P*	*d*
F3 vs. F4	52	−1.22	0.225	0.33	1.45	0.152	0.40
FC3 vs. FC4	52	−0.39	0.697	0.10	0.50	0.617	0.14
C3 vs. C4	52	−1.18	0.240	0.32	1.64	0.107	0.45
CP3 vs. CP4	52	−2.59	**0.012**	0.73	−0.93	0.354	0.25
F3 vs. C3	52	−5.84	** < .001**	1.61	−4.16	** < .001**	1.22
FC3 vs. CP3	52	−5.66	** < .001**	1.62	−2.59	**0.012**	0.70
F4 vs. C4	52	−5.06	** < .001**	1.40	−4.48	** < .001**	1.28
FC4 vs. CP4	52	−3.56	** < .001**	0.97	−1.27	0.208	0.34

Significant are in bold.

##### ERP amplitude before and after MIT

The ERP amplitude significantly increased after training for the two centrally located electrodes for the MIR task (FCz, *t*(26) = 2.97, *P* = 0.006, d = 0.06; Cz, t(26) = 2.31, *P* = 0.029, d = 0.05). There were no significant differences in ERP amplitude for the MIG task (*P* > 0.05; Fig. 6, Supplementary Fig. 1).

### ERP latency

Table 4 presents the mean values with SD of the ERP latencies for the electrodes of interest (Table 4). The repeated measures ANOVA has shown that the location of the electrodes and interaction between session and location factors had a statistically significant (*P* < 0.05) impact on the ERP latency parameter. The session and task had no significant effect on ERP latency (*P* > 0.05, Table 5).

**Table 4:** Mean value with standard deviation of ERP latency [ms] for motor imagery of reaching and motor imagery of grasping before and after 4 weeks of motor imagery training. Notice that the spatial arrangement of the electrodes in the table corresponds to the International 10–10 System.

Before training						After training					
Electrode	Mean ± SD [ms]	Electrode	Mean ± SD [ms]	Electrode	Mean ± SD [ms]	Electrode	Mean ± SD [ms]	Electrode	mean ± SD [ms]	electrode	mean ± SD [ms]
Motor imagery of reaching											
F3	373,4 ± 192,3	Fz	342,8 ± 156,2	F4	302,4 ± 160,2	F3	321,9 ± 150,9	Fz	338,0 ± 143,4	F4	341,8 ± 158,4
FC3	331,5 ± 147,8	FCz	380,7 ± 161,7	FC4	360,2 ± 227,8	FC3	336,9 ± 150,8	FCz	358,7 ± 138,9	FC4	409,5 ± 248,7
C3	332,8 ± 192,9	Cz	338,3 ± 164,5	C4	338,6 ± 188,8	C3	351,2 ± 215,1	Cz	375,0 ± 147,9	C4	407,9 ± 238,2
CP3	351,1 ± 233,0	CPz	492,9 ± 273,8	CP4	397,0 ± 265,8	CP3	444,9 ± 322,9	CPz	396,4 ± 214,3	CP4	279,5 ± 143,8

Electrode	Mean ± SD [ms]	Electrode	Mean ± SD [ms]	Electrode	Mean ± SD [ms]	Electrode	Mean ± SD [ms]	Electrode	Mean ± SD [ms]	Electrode	Mean ± SD [ms]
Motor imagery of grasping											
F3	344,6 ± 131,9	Fz	381,5 ± 151,9	F4	403,8 ± 199,4	F3	369,1 ± 194,1	Fz	377,2 ± 179,4	F4	417,5 ± 352,9
FC3	481,2 ± 260,4	FCz	413,5 ± 156,0	FC4	378,1 ± 203,3	FC3	364,4 ± 204,1	FCz	351,3 ± 139,7	FC4	328,0 ± 199,1
C3	386,6 ± 221,4	Cz	407,1 ± 179,7	C4	308,8 ± 198,3	C3	410,9 ± 240,9	Cz	392,2 ± 221,9	C4	375,9 ± 274,9
CP3	485,7 ± 363,5	CPz	347,7 ± 219,5	CP4	483,1 ± 408,5	CP3	515,8 ± 318,4	CPz	520,0 ± 345,8	CP4	333,2 ± 159,0

**Table 5:** The results of repeated measures ANOVA for ERP latency parameter.

Factor	*F* value	*P* value	${\upeta }_{\mathrm{P}}^{2}$
Session	*F*(1, 26) = 0.006	*P* = 0.940	0.001
Task	*F*(1, 26) = 3.283	*P* = 0.082	0.112
Location of the electrodes	*F*(5.506, 143.166) = 2.318	***P* = 0.041**	0.082
Session x task	*F*(1, 26) = 0.040	*P* = 0.844	0.002
Session x location	*F*(6.037, 156.952) = 2.557	***P* = 0.021**	0.090
Task x location	*F*(6.029, 156.752) = 1.701	*P* = 0.124	0.061
Session x task x location	*F*(5.069, 131.791) = 1.797	*P* = 0.117	0.065

Significants are in bold.

### ERP latency for different electrodes' locations

In the pairwise comparisons, there were no significant differences (*P* > 0.05) in ERP latencies between electrodes for the session before training (*P* > 0.05). However, the ERP latency for CP3 was significantly higher (*P* = 0.019) than that for CP4 for the MIR and MIG tasks (*P* < 0.001) for the session after training. The top-to-bottom comparison shows one significant difference. The ERP latency for FC4 compared to CP4 above the right hemisphere was significantly higher (*P* = 0.023) for the MIR task (Table 6).

**Table 6:** Results of pairwise comparison for ERP latency parameters between electrodes located above the right compared to the left hemisphere, and between electrodes located above the premotor cortex compared to the sensorimotor cortex of the same hemisphere for MIR and MIG before and after MIT assessed with independent *t*-*test*. Bold font indicates statistical significance.

Motor imagery of reaching							
Before					After		
Pair comparison	*df*	*t*	*P*	d	*t*	*P*	*d*
F3 vs. F4	52	−1.47	0.146	0.40	0.47	0.638	0.13
FC3 vs. FC4	52	0.55	0.585	0.15	1.29	0.200	0.36
C3 vs. C4	52	0.11	0.912	0.03	0.91	0.363	0.25
CP3 vs. CP4	52	0.67	0.503	0.18	−2.43	**0.019**	0.71
F3 vs. C3	52	0.77	0.442	0.21	−0.58	0.564	0.16
FC3 vs. CP3	52	−0.36	0.713	0.10	−1.57	0.121	0.45
F4 vs. C4	52	−0.76	0.451	0.20	−1.20	0.235	0.33
FC4 vs. CP4	52	−0.54	0.588	0.14	2.35	**0.023**	0.66

Motor imagery of grasping							
Before					After		
Pair comparison	*df*	*t*	*P*	d	*t*	*P*	*d*
F3 vs. F4	52	1.28	0.205	0.36	0.62	0.536	0.18
FC3 vs. FC4	52	−1.62	0.111	0.44	−0.66	0.510	0.18
C3 vs. C4	52	−1.36	0.180	0.37	−0.49	0.621	0.14
CP3 vs. CP4	52	−0.02	0.980	0.01	−2.66	**0.001**	0.77
F3 vs. C3	52	−0.84	0.402	0.23	−0.70	0.486	0.19
FC3 vs. CP3	52	−0.05	0.959	0.01	−2.08	0.042	0.58
F4 vs. C4	52	1.75	0.085	0.47	0.48	0.631	0.13
FC4 vs. CP4	52	−1.19	0.237	0.34	−0.10	0.917	0.03

Significants are in bold.

### ERP latency before and after MIT

**Figure 7:**
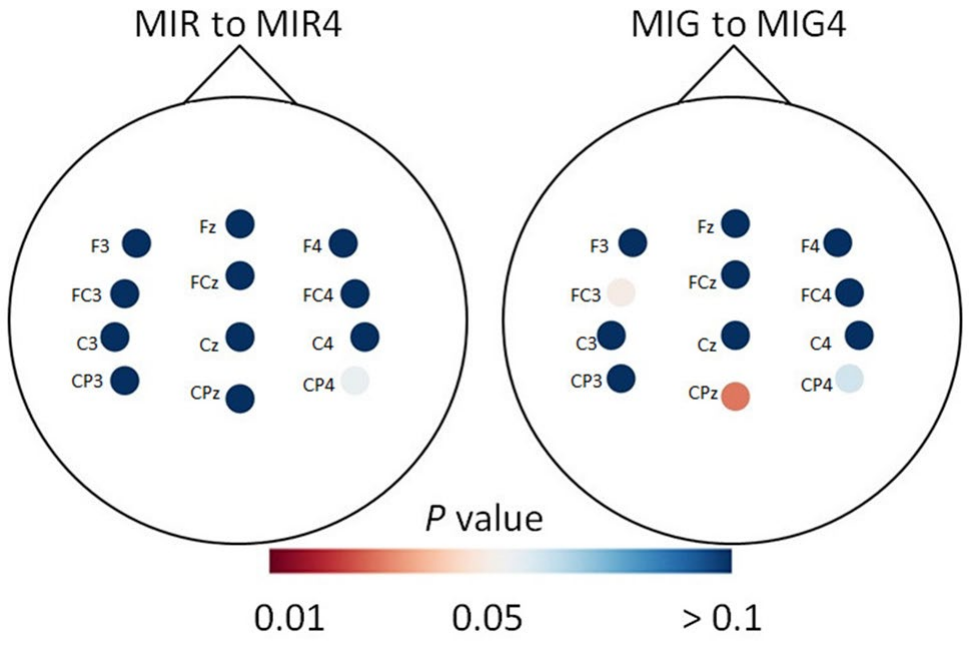
Heatmap depicting the *P* values of pairwise comparison for ERP latency between measurement sessions (before and after training) for motor imagery of reaching (MIR to MIR4) and motor imagery of grasping (MIG to MIG4).

ERP latency significantly increased after training in the CPz electrode (*t*(26) = −2.390, *P* = 0.024, d = 0.59) and decreased in the FC3 electrode (*t*(26) = 2.08, *P* = 0.047, d = 0.49) for the MIG task (Fig. 7). There were no differences (*P* > 0.05) in this parameter between sessions for the MIR task (Fig. 7, Supplementary Fig. 2).

### Correlation between the vividness of MI tasks and the EEG signal

The analysis shows that there were positive, moderate, and significant correlations between the VAS-MIR and ERP amplitude during the MIR task for the C4 (rho = 0.464, *P* = 0.015) and Fz (rho = 0.434, *P* = 0.024) electrodes and between VAS-MIR and ERP latency during the MIR task for the CPz electrode (rho = 0.433, *P* = 0.024) for the session before training. There were also positive, moderate, and significant correlations between the VAS-MIG and ERP amplitude during the MIG task for the session before training for the FCz (rho = 0.406, *P* = 0.036), Fz (rho = 0.460, *P* = 0.016), FC4 (rho = 0.584; *P* = 0.001), F4 (rho = 0.455, *P* = 0.017) and C4 (rho = 0.493, *P* = 0.009) electrodes. No significant correlation was found for the measurement session after MIT.

## Discussion

The study was focused on the effects of four weeks of kinesthetic MIT of functional goal-directed reaching, concerning objective cortical activity as determined by ERPs and subjective vividness of the imageries (VAS scale). We also determined the relationship between objective and subjective evaluations. Based on neurophysiological knowledge of the movement of the goal-directed reaching (reaching-to-grasp) in the classical approach^35^, we evaluated the effect of the training above while separately imagining the two components of this movement—reaching and grasping. Following this approach, we assessed whether the training affects both components of this movement similarly. We found that MIT influenced the EEG signal associated with reaching differently than that associated with grasping. The effect was more pronounced for the MIR than for the MIG. With a selection of 12 electrodes located in motor-related areas, we evaluated ERPs over the premotor and sensorimotor cortex of the right and left hemispheres, finding generally higher ERP amplitudes for the premotor cortices compared to the sensorimotor cortices in both sessions and tasks. In addition, significant correlations between the imageries' subjective vividness and the EEG were noted for the session before (not after) training. This finding means that the applied subjective assessment of imagining the tasks studied cannot be a simple, functional insight into the bioelectrical activity of the cerebral cortex, which changes over time due to training.

### ERP amplitude during MIR and MIG before training

Our results confirmed that the task (MIR and MIG) has an impact on the ERP amplitude. Higher values were noted for the MIR than the MIG task for two electrodes located above the contralateral hemisphere before MIT (one above the premotor cortex and one above the sensorimotor cortex). There were no significant differences for ten electrodes of interest, which partially overlaps with the results of Hétu et al.^55^. The authors showed that MI, regardless of the imagined parts of the body, usually involves many of the same brain structures. These structures mainly include the frontal lobe (on both sides in the area of the front frontal gyrus, the gyrus area of the pre-medial frontal gyrus, and the supplementary motor area), as was found in an activation likelihood estimation meta-analysis on fMRI and PET data.

Interestingly, there was no significant difference in the vividness of MI of the two tasks in our study. That is, after MIT, the difference increased but remained insignificant. This finding could be an effect of the similar neural network activation for MIR and MIG with the right upper limb. The study by Ofner et al.^24^ showed that upper limb movements, such as elbow flexion/extension, forearm supination/pronation, and hand opening/closing, with the dominant right upper limb, could be decoded from the time domain of the low-frequency EEG signal. They analyzed ERPs related to the above movements and the kinesthetic MI of the same actions. They noted that ERPs from motor execution (ME) were more pronounced than ERPs from similar MI tasks. The authors concluded that different movements of the same upper limb could be decoded from ERPs and differentiated against each other, with promising accuracies for the ME classification and relatively low accuracies for the MI tasks. Thus, they also indirectly indicated similar characteristics of ERPs from different MIs of upper limb tasks. They pointed out that training can be beneficial in the context of MI task discrimination by ERPs.

### ERP amplitude during MIR and MIG after training

In our study, the ability to discriminate imagined reaching from imagined grasping increased after training. Higher ERP amplitudes relating more to MIR than MIG were noted for one central electrode (Fz) and three electrodes above the ipsilateral hemisphere (two above the premotor and one above the sensorimotor cortex). A repeated measures ANOVA also confirmed that the interaction between the session (before vs. after training), the task, and the electrode location impacted ERP amplitude. It means that the distinction between tasks in terms of ERPs amplitude increased after MIT. Therefore, it can be assumed that the classification accuracy would increase after MIT for the mentioned electrodes, but it was not checked in our study. Higher ERP amplitude for the electrodes located above the regions of the ipsilateral (right) hemisphere for the MIR task than for the MIG task may reflect the involvement of these structures in the programming of the reaching movement trajectory, as was shown by the study by Binkofski^56^. These authors showed that the brain's right hemisphere of the indicated areas dominates in the context of spatial movement characteristics. This finding may explain the higher ERP amplitudes of the MIR relative to the MIG after four weeks of MIT in our study. Our results also showed that MIT had a higher effect on MIR than MIG. This finding could be related to the instruction given to participants during motor execution (ME) of reaching to grasp a book before each of the MIT. We asked the subjects to pay attention to kinesthesia during reaching and the sensation of grasping when in contact with the imagined book (at the end of the rehearsal of this movement). Although not tested, participants may have spent more time imagining reaching than grasping. This could partially explain the results obtained in a pairwise comparative analysis between sessions for which a significant increase in ERP amplitude was found for two central electrodes for the MIR task and the finding of no differences in this parameter for the MIG task. Significantly higher ERP amplitudes were noted for electrodes placed over the supplementary motor cortex (FCz and Cz), which is highly involved in imagining movements, as indicated by the studies of many other authors^5,55,57,58^. According to Sobierajewicz et al.^12^, the increase in ERP amplitude in the context of imagining movement corresponds to the process of learning this task. Therefore, the significant increase in amplitude for the indicated electrodes for MIR in our study confirms a greater contribution of mental goal-directed reaching to the reaching component than to the grasping component. The indicated learning process is confirmed by the psychometric test results obtained. The ability to imagine the tested tasks increased significantly after MIT.

### ERP amplitude and ERP latency for different cortical regions

Our results showed that the location of the electrodes had a significant impact on both analyzed parameters of the EEG signal. Higher ERP amplitudes (approximately double) were noted for all electrodes located above the premotor cortices compared to those above the sensorimotor cortices of the same cerebral hemisphere for both tasks and sessions. It could be related to the dynamic functions of the premotor cortices and their bigger involvement in MI tasks compared to the sensorimotor regions. It corresponds to previous studies showing that premotor regions play a critical role in generating mental images^5,58^. A graph-theory analysis of connectivity during MI made by Xu et al.^59^ identified the premotor cortex as the crucial node of MI. Higher ERP amplitudes for electrodes located above premotor cortices than those located above sensorimotor cortices could be related to the extra involvement of frontal executive processes during MI compared to ME, as was shown by Van der Lubbe et al.^60^. The authors indicated a few differences between MI and ME, including increased activity of sensorimotor areas in the case of ME as compared to MI, which corresponds to our results in part (although we did not compare ERP with MRCP related to ME). They also suggested that visuospatial attention is reduced in the case of MI relative to ME and their results support the motor-cognitive and not the functional equivalence model of MI.

Additionally, in our study, hemispherical differences were noted only for the CP3 electrode with respect to the CP4 electrode. Other hemispheric differences were not noted. This means that there is no significant difference between contralateral and ipsilateral regions' involvement in those tasks assessed by the time-domain EEG signal analysis. It corresponds to other studies that showed the bilateral involvement of different brain structures in unilateral MI tasks^55^. The meta-analysis looking at the general pattern of neural activation during the general MI of Hétu et al.^55^ showed several large clusters spanning both hemispheres. Areas identified included bilateral inferior frontal gyri, the SMA, the bilateral superior parietal lobules, and others. The imaging findings cited above could be translated to the EEG signal analyzed in the time domain. Ofner et al.^24^ showed that ERP patterns from MI were more centrally located than the more lateralized MRCP pattern from ME.

### Correlation between the vividness of MIR and MIG and EEG signal

In our study, significant, moderate, positive correlations between the subjective vividness of imageries and the EEG signal were noted for the ERP amplitude for both tasks for the session before training. They were shown for electrodes above the ipsilateral sensorimotor cortex, the Fz electrode located centrally above the frontal cortex, and electrodes above the ipsilateral premotor cortex for the MIG task (not for the MIR task). The ERP amplitude is assumed to reflect the number of active neurons, their synchronization, and discharge rates^27^. Thus, the results could indicate that the activity of these brain areas intensified during unfamiliar imagery tasks before training. The ERP could also reflect the potential inhibition of motor activity^34,61^ in MI tasks, as subjects were asked to imagine and withheld or did not perform the tasks. Previous research by Zabicki et al.^43^ revealed a link between the subjective impression of MI vividness of right-hand actions and the objective neural activity pattern during the corresponding MI tasks assessed by fMRI. They found a significant positive correlation between the left ventral premotor cortex and the right inferior parietal lobe. They concluded that these regions particularly reflect perceived imagery vividness. Our research showed correlations between vividness and the EEG signal for electrodes above other cortical regions. Still, these results are difficult to compare due to the different objective methods used, which examine different features of brain function. Moreover, Zabicki et al.^43^ added that imagined tasks with higher vividness ratings are significantly more distinguishable within the areas mentioned above, suggesting that a vivid motor image correlates with a more distinct neural representation. In our study, the perceived vividness of MI tasks increased significantly after training. Also, there was no significant correlation between the subjective impression of MI vividness and the objective EEG signal for the measurement session (after MIT). It could mean that higher vividly imagined motor tasks after MIT elicit reduced single electrode-specific cortical activity that could not be captured by ERPs (opposite to results by fMRI in Zabicki et al.^43^). In other words, our results have shown that use-dependent changes in the vividness of imagination of motor reaching and grasping, as well as cortical activity during these tasks in terms of ERPs, as the effect of MIT alter these relationships.

Further studies are needed on the associations between objective methods and psychometric tests that are easy to use in clinical or home environments. Finding such correlates could help control the course of imagining in patients.

### Limitations of the study

Some limitations must be pointed out. First, a within-subjects design was a type of experimental study used with no control group. Therefore, to avoid the impact of possible confounding factors other than the MIT on the parameters analyzed, we ensured that all the interventions (training and sessions) were conducted similarly. According to Slagter et al.^62^, this study design provides optimal power to identify mental training-related changes. This finding reflects that within-group variability tends to be smaller than across-subject variability, as in the case of the second fundamental strategy for examining the effects of mental training—cross-sectional ones^62^. The reliability of our study is also supported by the sample size and the statistical tests performed. Secondly, since MI of reaching and grasping were repeated 20 times during both measurement sessions, it is challenging to exclude central adaptations due to task repetition. However, this factor is hard to eliminate due to the standards of the EEG protocol with its time-domain analysis. Furthermore, the tasks studied were not selected in a random order, which potentially constitutes another study limitation and may contribute to the 'order effect.' However, the measurement sessions were conducted the same way. Also, the order of the tasks was the same for all participants before and after training. This sequence corresponds to the natural course of the reach-to-grasp movement, according to an assumed model of this movement^39^.

### Conclusions

In this paper, we show the effect of kinesthetic MIT of goal-directed reaching on MI of the two components of this movement (according to the classical approach of neural control of this goal-directed movement), that is, reaching and grasping with the dominant upper limb in healthy young participants, and the subjective vividness of those tasks. We conclude that this training influences reaching and grasping differently. The effect was more pronounced for the imagery of reaching than for grasping. For both tasks and sessions, higher ERP amplitudes were noted for electrodes above the premotor than the sensorimotor cortices. We also showed that use-dependent changes in the ability to MIR and MIG and the cortical activity during those tasks (in terms of ERPs) alter the correlation between the two. Hence, the applied subjective evaluation of imagining the tasks cannot be a simple, functional insight into the bioelectrical activity of the cerebral cortex expressed by the ERPs in the process of mental training.

Our findings are relevant to neurorehabilitation and goal-directed reaching by mental training. The changes we noted in ERPs after training may benefit the use of non-invasive EEG in the context of BCI.

## Supplementary Information

Supplementary Information.

## Data Availability

The datasets generated and/or analysed during the current study are not publicly available due to ethical restrictions but are available from the corresponding author on reasonable request.
