# Supplementary material for: Motor imagery training of goal-directed reaching in relation to imagery of reaching and grasping in healthy people
Source: Sci Rep. 2022 Nov 3;12:18610. doi: 10.1038/s41598-022-21890-1 (PMC9633838; doi:10.1038/s41598-022-21890-1)

Supplementary Figure 1. Results of the comparison between sessions (before to after) for ERP amplitude during motor imaginary of reaching assessed with a paired-samples *t*-test (only for electrodes with significant differences).

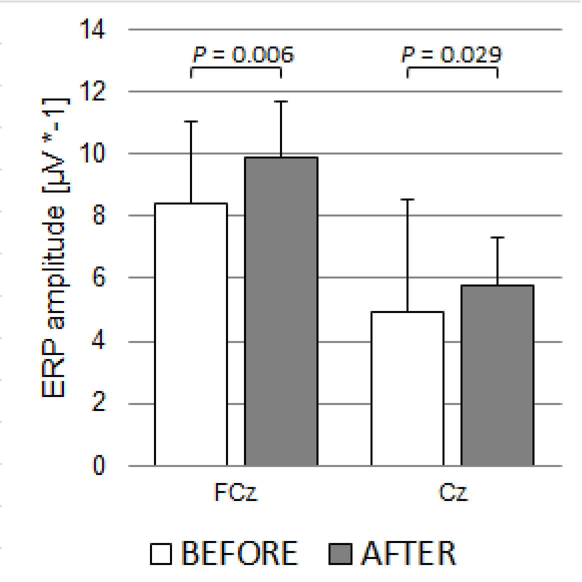

Supplementary Figure 2. Results of the comparison between sessions (before to after) for ERP latency during motor imaginary of grasping assessed with a paired-samples *t*-test (only for electrodes with significant differences).

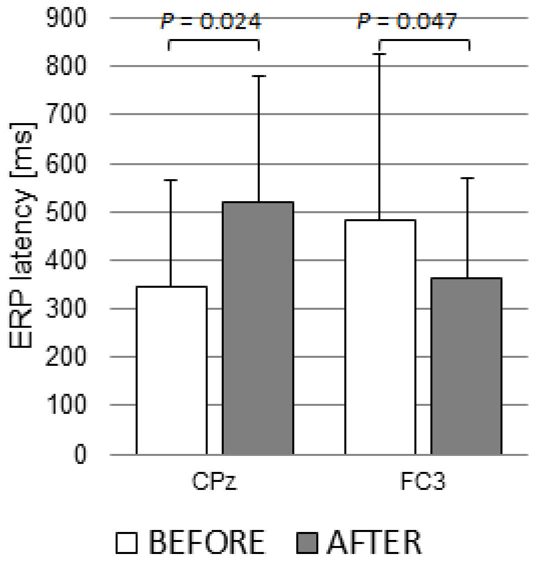

Supplement: Supplementary file 1 — Supplementary Information. [file 41598_2022_21890_MOESM1_ESM.pdf]
